# Supplementary material for: Heat adaptation of phage T7 under an extended genetic code
Source: Virus Evol. 2021 Dec 1;7(2):veab100. doi: 10.1093/ve/veab100 (PMC8923235; doi:10.1093/ve/veab100)
Supplement: veab100_Supp [file veab100_supp.zip › Sequencing_Analysis.zip!/Sequencing_Analysis/S1/evidence/MC_SIDE_1_38.html]

BRESEQ :: Evidence


| Predicted mutation | | | | | | |
| --- | --- | --- | --- | --- | --- | --- |
| evidence | seq id | position | mutation | annotation | gene | description |
| MC JC | NC\_001604.1 | 13,569 | Δ770 bp | intergenic (–/–) | – / – | –/– |

| Missing coverage evidence... | | | | | | | | | | |
| --- | --- | --- | --- | --- | --- | --- | --- | --- | --- | --- |
|  |  |  | seq id | start | end | size | ←reads | reads→ | gene | description |
| \* | \* | ÷ | NC\_001604.1 | 13569 | 14338 | 770 | 4673 [104] | [4] 4561 | *–/–* | –/– |

| New junction evidence | | | | | | | | | | | |
| --- | --- | --- | --- | --- | --- | --- | --- | --- | --- | --- | --- |
|  | | seq id | position | reads (cov) | reads (cov) | score | skew | freq | annotation | gene | product |
| \* | ? | NC\_001604.1 | = 13568 | 104 (0.020) | 4361 (1.060) | 369/586 | 0.2 | 98.8% | intergenic (–/–) | *–/–* | –/– |
|  | ? | NC\_001604.1 | 14339 = | 1 (0.000) |  |  |  |  | intergenic (–/–) | *–/–* | –/– |

Only 100 of 4673 total aligned reads displayed.  

`GGATTCTTGATGACTTTCCAGACGACTACGAGAAGTTTCGCTGGAGAGTCCCATTCTAATACGACTCACTAAAGGAGACACACCATGTTCAAACTGATTAAGAAGTTAGGCCAACTGCTGGTTCGTATGTACAACGTGGAAGCCAAGCGACTGAACGATGAGGCTCGTAAAGAGGCCACACAGTCACGCGCTCTGGCGATTCGCTCCAACGAACTGGCTGACAGTGCATCCACTAAAGTTACCGAGGCTGCCCGTGTGGCAAACCAAGCTCAACAGCTTTCCAAATTCTTTGAGTAAT‑C‑AAACAGGAGAAACCATTATGTCTAACGTAGCTGAAACTATCCGTCTATCCGATACAGCTGACCAGTGGAACCGTCGAGTCCACATCAACGTTCGCAACGGTAAGGCGACTATGGTTTACCGCTGGAAGGACTCTAAGTCCTCTAAGAATCACACTCAGCGTATGACGTTGACAGATGAGCAAGCACTGCGTCTGGTCAATGCGCTTACCAAAGCTGCCGTGACAGCAATTCATGAAGCTGGTCGCGTCAATGAAGCTATGGCTATCCTCGACAAGATTGATAACTAAGAGTGGTATCCTCAA  >  NC_001604.1/13268‑13868`  

                                                                                                                                                                                                                                                                                                              `|`                                                                                                                                                                                                                                                                                                              
`ggATTCTTGATGACTTTCCAGACGACTACGAGAAGTTTCGCTGGAGAGTCCCATTCTAATACGACTCACTAAAGGAGACACACCATGTTCAAACTGATTAAGAAGTTAGGCCAACTGCTGGTTCGTATGTACAACGTGGAAGCCAAGCGACTGAACGATGAGGATCGTAAAGAGGCCACACAGTCACGCGCTCTGGCGATTCGCTCCAACGAACTGGCTGACAGTGCATCCACTAAAGTTACCGAGGCTGCCCGGGTGGCAAACCAAGCTCAACAGCTTTCCAAATTCTTTGAGTAAT‑C‑AA                                                                                                                                                                                                                                                                                                              >  1:424686‑M1/1‑301 (MQ=255)`  
    `tCTTGATTACTTTCCAGACGACTACGAGAAGTTTCGCTGGAGAGTCCCATTCTAATACGACTCACTAAAGGAGACACACCATGTTCAAACTGATTAAGAAGTTAGGCCAACTGCTGGTTCGTATGTACAACGTGGAAGCCAAGCGACTGAACGATGAGGCTCGTAAAGAGGCCACACAGTCACGCACTCTGGCGATTCGCTCCAACGAACTGGCTGACAGTGCATCCACTAAAGTTACCGAGGCTGCCCGTGTGGCAAACCAAGCTCAACAGCTTTCCAAATTCTTTGAGTAAT‑C‑AAtagg                                                                                                                                                                                                                                                                                                          <  1:110685‑M1/301‑5 (MQ=255)`  
    `tCTTGATGACTTTCCAGACGACTACGCTAAGTTTCGCTGGAGAGTCCCATTCTAATACGACTCACTAAAGGAGACACACCATGTTCAAACTGATTAAGAAGTTAGGCCAACTGCTGGTTCGTATGTACAACGTGGAAGCCAAGCGACTGAACGATGAGGCTCGTAAAGAGGCCACACAGTCACGCGCTCTGGCGATTCGCTCCAACGAACTGGCTGACAGTGCATCCACTAAAGTTACCGAGGCTGCCCGTGTGGCAAACCAAGCTCAACAGCTTTCCAAATTCTTTGAGTAAT‑C‑AAtagg                                                                                                                                                                                                                                                                                                          <  1:311041‑M1/301‑5 (MQ=255)`  
               `ttCCAGACGACTACGAGAAGTTTCGCTGGAGAGTCCCATTCTAATACGACTCACTAAAGGAGACACACCATGTTCAAACTGATTAAGAAGTTAGGCCAACTGCTGGTTCGTATGTACAACGTGGAAGCCAAGCGACTGAACGATGAGGCTCGTAAAGAGGCCACACAGTCACGCGCTCTGGCGATTCGCTCCAACGAACTGGCTGACAGTGCATCCACTAAAGTTACCGAGGCTGCCCGTGTGGCAAACCAAGCTCAACAGCTTTCCAAATTCTTTGAGTAAT‑C‑AAtaggagaaatcaata                                                                                                                                                                                                                                                                                               >  1:389712‑M1/1‑286 (MQ=255)`  
                    `gagatgtgtagaagagacagctggagagtcccattctaaTACGACTCACTAAAGGAGACACACCATGTTCAAACTGATTAAGAAGTTAGGCCAACTGCTGGTTCGTATGTACAACGTGGAAGCCAAGCGACTGAACGATGAGGCTCGTAAAGAGGCCACACAGTCACGCGCTCTGGCGATTCGCTCCAACGAACTGGCTGAAAGTGCATCCACTAAAGTTACCGAGGCTGCCCGTGTGGCAAACCAAGCTCAACAGCTTTCCAAATTCTTTGAGTAAT‑CAAAcaggagaaatcaatatgat                                                                                                                                                                                                                                                                                           <  1:334219‑M1/282‑20 (MQ=255)`  
                    `gacgacTACGAGAAGTTTCGCTGGAGAGTCCCATTCTAATACGACGCACTAAAGGAGACACACCATGTTCAAACTGATTAAGAAGTTAGGCCAACTGCTGGTTCGTATGTACAACGTGGAAGCCAAGCGACTGAACGATGAGGCTCGAAAAGAGGCCACACAGTCACGCGCTATGGCGATTCGCTCCAACGAACTGGCTGACAGTGCATCCACTAAAGTTACCGAGGCTGCCCGTGTGGCAAACCAAGCTCAACAGCTTTCCAAATTCTTTGAGTAAT‑CAAAcaggagaaatcaatatgat                                                                                                                                                                                                                                                                                           <  1:429904‑M1/301‑20 (MQ=255)`  
                         `cTACGAGAAGTTTCGCTGGAGAGTCCCATTCTAATACGACTCACTAAAGGAGACACACCATGTTCAAACTGATTAAGAAGTTAGGCCAACTGCTGGTTCGTATGTACAACGTGGAAGCCAAGCGACTGAACGATGAGGCTCGTAAAGAGGCCACACAGTCACGCGCTCTGGCGATTCGCTCCAACGAACTGGCTGACAGTGCATCCACTAAAGTTACCGAGGCTGCCCGTGTGGCAAACCAAGCTCAACAGCTTTCCAAATTCTTTGAGTAAT‑CAAAcaggagaaatcaatatgatcgttt                                                                                                                                                                                                                                                                                      >  1:100160‑M1/1‑277 (MQ=255)`  
                           `aCGAGAAGTTTCGCTGGAGAGTCCCATTCTAATACGACTCACTAAAGGAGACACACCATGTTCAAACTGATTAAGAAGTTAGGCCAACTGCTGGTTCGTATGTACAACGTGGAAGCCAAGCGACTGAACGATGAGGCTCGTAAAGAGGCCACACAGTCACGCGCTCTGGCGATTCGCTCCAACGAACTGGCTGACAGTGCATCCACTAAAGTTACCGAGGCTGCCCGTGTGGCAAACCAAGCTCAACAGCTTTCCAAATTCTTTGAGTAAT‑C‑AAACAGGAGAAACCATTATGACtgtctct                                                                                                                                                                                                                                                                                   >  1:197407/1‑295 (MQ=255)`  
                              `agaAGTTTCGCTGAAGAGTCACATTCTAATACGACTCACTAAAGGAGACACACCAGGTTCAAACTGATTAAGAAGTTAGGCCAGCTGCTGGTTCGTATGTACAACGTGGAAGCCAAGCGACTGAACGATGAGGCTCGTAAAGAGGCCACACAGTCACGCGCTCTGGCGATTCGCTACAACGAACTGGCTGACAGTGCATCCACTAAAGTTACCGAGGCTGCCCGTGTGGCAAACCAAGCTCAACAGCTTTCCAAATTCTTTGAGTAAT‑C‑AAACAGGAGAAACCATTATgatcgtttctgac                                                                                                                                                                                                                                                                                <  1:172187/301‑13 (MQ=255)`  
                                `gacagtcgctgGAGAGTCCCATTCTAATACGACTCACTAAAGGAGACACACCATGTTCAAACTGATTAAGAAGTTAGGCCAACTGCTGGTTCGTATGTACAACGTGGAAGCCAAGCGACTGAACGATGAGGCTCGTAAAGAGGCCACACAGTCACGCGCTCTGGCGATTCGCTCCAACGAACTGGCTGACAGTGCATCCACTAAAGTTACCGAGGCTGCCCGTGTGGCAAACCAAGCTCAACAGCTTTCCAAATTCTTTGAGTAAT‑C‑AAtaggagaaatcaatatgatcgtttctgacatc                                                                                                                                                                                                                                                                              <  1:558171‑M1/296‑33 (MQ=255)`  
                                      `cGCTGGAGAGTCCCATTCTAATACGACTCACTAAAGGAGACACACCATGTTCAAACTGATTAAGAAGTTAGGCCAACTGCTGGTTCGTATGTACAACGTGGAAGCCAAGCGACTGAACGATGAGGCTCGTAAAGAGGCCACACAGTCACGCGCTCTGGCGATTCGCTCCAACGAACTGGCTGACAGTGCATCCACTAAAGTTACCGAGGCTGCCCGTGTGGCAAACCAAGCTCAACAGCTTTCCAAATTCTTTGAGTAAT‑CAAAcaggagaaatcaatatgatcgtttctgacatcgaagc                                                                                                                                                                                                                                                                         >  1:65375‑M1/1‑264 (MQ=255)`  
                                               `gTCCCATTCTAATACGACTCACTAAAGGAGACACACCATGTTCAAACTGATTAAGAAGTTAGGCCAACTGCTGGTTCGTATGTACAACGTGGAAGCCAAGCGACTGAACGATGAGGCTCGTAAAGAGGCCACACAGTCACGCGCTCTGGCGATTCGCTCCAACGAACTGGCTGACAGTGCATCCACTAAAGTTACCGAGGCTGCCCGTGTGGCAAACCAAGCTCAACAGCTTTCCAAATTCTTTGAGTAAT‑CAAAcaggagaaatcaatatgatcgtttctgacatcgaagctaacgccct                                                                                                                                                                                                                                                                >  1:610084‑M1/1‑255 (MQ=255)`  
                                                    `gttcTAATACGACTCACTAAAGGAGACACACCATGTTCAAACTGATTAAGAAGTTAGGCCAACTGCTGGTTCGTATGTACAACGTGGAAGCCAAGCGACTGAACGATGAGGCTCGTAAAGAGGCCACACAGTCACGCGCTCTGGCGATTCGCTCCAACGAACTGGCTGACAGTGCATCCACTAAAGTTACCGAGGCTGCCCGTGTGGCAAACCAAGCTCAACAGCTTTCCAAATTCTTTGAGTAAT‑CAAAcaggagaaatcaatatgatcgtttctgacatcgaagctaacgccctcttag                                                                                                                                                                                                                                                           <  1:477622‑M1/300‑52 (MQ=255)`  
                                                     `ttCTAATACGACTCACTAAAGGAGACACACCATGTTCAAACTGCTTAAGAAGTTAGGCCAACTGCTGGTTCGTATGTACAACGTGGAAGCCAAGCGACTGAACGATGAGGCTCGTAAAGAGGACACACAGTCACGCGCTCTGGAGATTCGCTCCAACGTACTGGCTGACAGTGCATCCACTAAAGTTACCGAGGCTGCCCGTGTGGCAAACCAAGCTCAACAGCTTTCCAAATTCTTTGAGTAAC‑C‑ACtaggagaaatcactatgatcgtttctgcaatcgcagctaacgacctcatagat                                                                                                                                                                                                                                                         >  1:315700‑M1/1‑248 (MQ=255)`  
                                                     `ttCTAATACGACTCACTAAAGGAGACACACCATGTTCAAACTGATTAAGAAGTTAGGCCAACTGCTGGTTCGTATGTACAACGTGGAAGCCAAGCGACTGAACGATGAGGCTCGTAAAGAGGCCACACAGTCACGCGCTCTGGCGATTCGCTCCAACGAACTGGCTGACAGTGCATCCACTAAAGTTACCGAGGCTGCCCGTGTGGCAAACCAAGCTCAACAGCTTTCCAAATTCTTTGAGTAAT‑C‑AAtaggagaaatcaatatgatcgtttctgacatcgaagctaacgccctcttagag                                                                                                                                                                                                                                                         <  1:273906‑M1/301‑54 (MQ=255)`  
                                                     `ttCTAATACGACTCACTAAAGGAGACACACCATGTTCAAACTGATTAAGAAGTTAGGCCAACTGCTGGTTCGTATGTACAACGTGGAAGCCAAGCGACTGAACGATGAGGCTCGTAAAGAGGCCACACAGTCACGCGCTCTGGCGATTCGCTCCAACGAACTGGCTGACAGTGCATCCACTAAAGTTACCGAGGCTGCCCGTGTGGCAAACCAAGCTCAACAGCTTTCCAAATTCTTTGAGTAAT‑C‑AAtaggagaaatcaatatgatcgtttctgacatcgaagctaacgccctcttagag                                                                                                                                                                                                                                                         >  1:165482‑M1/1‑248 (MQ=255)`  
                                                     `ttCTAATACGACTCACTAAAGGAGACACACCATGTTCAAACTGATTAAGAAGTTAGGCCAACTGCTGGTTCGTATGTACAACGTGGAAGCCAAGCGACTGAACGATGAGGCTCGTAAAGAGGCCACACAGTCACGCGCTCTGGCGATTCGCTCCAACGAACTGGCTGACAGTGCATCCACTAAAGTTACCGAGGCTGCCCGTGTGGCAAACCAAGCTCAACAGCTTTCCAAATTCTTTGAGTAAT‑C‑AAtaggagaaataaatatgatcgtttctgacatcgaagctaacgccctcttagag                                                                                                                                                                                                                                                         >  1:484040‑M1/1‑248 (MQ=255)`  
                                                     `ttCTAATACGACTCACTAAAGGAGACACACCATGTTCAAACTGATTAAGAAGTTAGGCCAACTGCTGGTTCGTATGTACAACGTGGAAGCCAAGCGACTGAACGATGAGGCTCGTAAAGAGGCCACACAGTCACGCGCTCTGGCGATTCGCTCCAACGAACTGGCTGACAGTGCATCCACTAAAGTTACCGAGGCTGACCGTGTGGCAAACCAAGCTCAACAGCTTTCCAAATTCTTTGATTAAT‑CAAAcaggagaaatcaatatgagcgtttctgccatcgaagctaccgccctcttaga                                                                                                                                                                                                                                                          >  1:347750‑M1/1‑249 (MQ=255)`  
                                                     `ttCTAATACGACTCACTAAAGGAGACACACCATGTTCAAACTGATTAAGAAGTTAGGCCAACTGCTGGTTCGTATGTACAACGTGGAAGCCAAGCGACTGAACGATGAGGCTCGTAAAGAGGCCACACAGTCACGCGCTCTGGCGATTCGCTCAAACGAACTGGCTGACAGTGCATCCACTAAAGTTACCGAGGCTGCCCGTGTGGCAAACCAAGCTCAACAGCTTTCCAAATTCTTTGAGTAAT‑C‑AAtaggagaaatcaatatgatcgtttctgacatcgaagctaacgccctcttagat                                                                                                                                                                                                                                                         <  1:93‑M1/301‑54 (MQ=255)`  
                                                     `gtgggctcggagatgtgtataagagacagccatgttcaaactgattaagaagttaggccaACTGCTGGTTCGTATGTACAACGTGGAAGCCAAGCGACTGAACGATGAGGCTCGTAAAGAGGCCACACAGTCACGCACTCTGGCGATTCGCTCCAACGAACTGGCTGACAGTGCATCCACTAAAGTTACCGAGGCTGCCCGTGTGGCAAACCAAGCTCAACAGCTTTCCAAATTCTTTGAGTAAT‑C‑AAtaggagaaatcaatatgatcgtttctgacatcgaagctaacgccctcttagag                                                                                                                                                                                                                                                         <  1:569445‑M1/272‑54 (MQ=255)`  
                                                     `agagaaagcgactcactAAAGGAGACACACTATGTTCAAACTGATTAAGAAGTTAGGCCAACTGCTGGTTCGTAGGTACAACGTGGAAGCCAAGCGACTGAACGATGAGGCTCGTAAAGAGGCCAAACAGTCACGCGCGCTGGCGATTCGCTCCAACGAACTGGCTGACAGTGCATCCACTAAAGTTACCGAGGCTGCCCGTGTGGCAAACCAAGCTCAACAGCTTTCCAAATTCTTTGAGTAAT‑C‑AAtaggagaaatcaatatgatcgtttctgacatcgaagctaacgccctcttagag                                                                                                                                                                                                                                                         <  1:412337‑M1/293‑54 (MQ=255)`  
                                                      `tCTAATACGACTCACTAAAGGAGACACACCATGTTCAAACTGATTAAGAAGTTAGGCCAACTGCTGGTTCGTATGTACAACGTGGAAGCCAAGCGACTGAACGATGAGGCTCGTAAAGAGGCCACACAGTCACGCGCTCTGGCGATTCGCTCCAACGAACTGGCTGACAGTGCATCCACTAAAGTTACCGAGGCTGCCCGTGTGGCAAACCAAGCTCAACAGCTTTCCAAATTCTTTGAGTAAT‑CAAAcaggagaaatcaatatgatcgtttctgacatcgaagctaacgccctcttagag                                                                                                                                                                                                                                                         <  1:215407‑M1/301‑54 (MQ=255)`  
                                                      `actAATACGACTCACTAAAGGAGACACACCATGTTCAAACTGATTAAGAAGTTAGGCCAACTGCTGGTTCGGATGTACAACGTGGAAGCCAAGCGACTGAACGATGAGGCTCGTAAAGAGGCCACACAGTCACGCGCTCTGGCGATTCGCTCCAACGAACTGGCTGACAGTGCATCCACTAAAGTTACCGAGGCTGCCCGTGTGGCAAACCAAGCTCAACAGCTTTCCAAATTCTTTGAGTAAT‑CAAAcaggagaaaccattatgatcgtttctgacatcgaagctaacgccctcttagag                                                                                                                                                                                                                                                         <  1:52508‑M1/300‑54 (MQ=255)`  
                                                      `aaaaagagacagaactaaaggagacacACCATGTTCAAACTGATTAAGAAGGTAGGCCAACTGCTGGTTCGGATGTACAACGTGGAAGCCAAGCGACTGAACGATGAGGGTCGTAAAGAGGCCACACAGTCACGCGATCTGGCGATTCGCTCCAAAGAACTGGCTGACAGTGCATCCACTAAAGTTACCGAGGCTGCCCGTGTGGCAAACCAAGAACAACAGCTTTCCAAATTCTTTGAGTAAT‑CAAAcaggagaaaccattatgatcgtttctgacatcgaagctaacgccctcttagag                                                                                                                                                                                                                                                         <  1:543210‑M1/288‑54 (MQ=255)`  
                                                       `cTAATACGACTCACTAAAGGAGACACACCATGTTCAAACTGATTAAGAAGTTAGGCCAACTGCTGGTTCGTATGTACAACGTGGAAGCCAAGCGACTGAACGATGAGGCTCGTAAAGAGGCCACACAGTCACGCGCTCTGGCGATTCGCTCCAACGAACTGGCTGACAGTGCATCCACTAAAGTTACCGAGGCTGCCCGTGTGGCAAACCAAGCTCAACAGCTTTCCAAATTCTTTGAGTAAT‑C‑AAtaggagaaatcaatatgatcgtttctgacatcgaagctaacgccctcttagagag                                                                                                                                                                                                                                                       >  1:466165‑M1/1‑246 (MQ=255)`  
                                                       `cTAATACGACTCACTAAAGGAGACACACCATGTTCAAACTGATTAAGAAGTTAGGCCAACTGCTGGTTCGTATGTAAAACGTGGAAGCCAAGCGACTGAACGATGAGGCTCGTAAAGAGGCCACACAGTCACGCGCTCTGGCGATTCGCTCCAACGAACTGGCTGACAGTGCATCCACTAAAGTTACCGAGGCTGCCCGTGTGGCAAACCAAGCTCAACAGCTTTCCAAATTCTTTGAGTAAT‑C‑AAtaggagaaatcaatatgatcgtttctgacatcgaagctaacgccctcttagagag                                                                                                                                                                                                                                                       <  1:381097‑M1/301‑56 (MQ=255)`  
                                                               `ataagagacaggagacagaACATGTTCAAACTGATTAAGAAGTTAGGCCAACTGCTGGTTCGTATGTACAACGTGGAAGCCAAGCGACTGAACGATGAGGCTCGTAAAGAGGCCAAACAGTCACGCGCTCTGGCGATTTGCGCCAACGAACTGGCTGAAAGTGCATCCACTAAAGTTACCGAGGCTGCCCGTGTGGCAAACCAAGCTCAACAGCTTTCCAAATTCTTTGAGTAAT‑C‑AAtaggagaaatcaatatgatcgtttctgacatcgaagctaacgccctcttagagagcgtcacta                                                                                                                                                                                                                                               <  1:400870‑M1/292‑64 (MQ=255)`  
                                                               `aCTCACTAAAGGAGACAAACCAGGTTCAAACTGATTAAGAAGTTAGGCCAACTGCTGGTTCGTATGTACAACGTGGAAGCCAAGCGACTGAACGATGAGGCTCGTAAAGAGGCCACACAGTCACGCGCTCTGGCGATTCGCTCCAACGAACTGGCTGACAGTGCATCCACTAAAGTTACCGAGGCTGCCCGTGTGGCAAACCAAGCTCAACAGCTTTCCAAATTCTTTGAGTAAT‑C‑AAtaggagaaatcaatatgatcgtttctgacatcgaagctaacgccctcttagagagcgtcacta                                                                                                                                                                                                                                               <  1:86112‑M1/301‑64 (MQ=255)`  
                                                                  `gagagggggataaggaagaggttaaaacagagtaagcagttAGGCCAACTGCGGGTTAGTATGTAAAACGTGGAAGCCAAGCGACTGAACGATGAGGCTCGAAAAGAGGCCACACAGTCACGCGCTCTGGAGATTCGCTCCAACGAACTGGCTGACAGTGCATACACTAAAGTTACCGAGGCTGCCCGTGTGGCAAACCAAGCTCAACAGCTTTCCAAATTCTTTGAGTAAT‑C‑AAtaggagaaataaatatgatcgtttatgacatcgaagctaacgccctcttagagagcgtcactaagt                                                                                                                                                                                                                                            <  1:376617‑M1/281‑67 (MQ=255)`  
                                                                  `cACTAAAGGAGACACACCATGTTCAAACTGATTAAGAAGTTAGGCCAACTGCTGGTTCGTATGTACAACGTGGAAGCCAAGCGACTGAACGATGAGGCTCGTAAAGAGGCCACACAGTCACGCGCTCTGGCGATTCGCTCCAACGAACTGGCTGACAGTGCATCCACTAAAGTTACCGAGGCTGCCCGTGTGGCAAACCAAGCTCAACAGCTTTCCAAATTCTTTGAGTAAT‑CAAAcaggagaaatcaatatgatcgtttctgacatcgaagctaacgccctcttagagctgtctcttata                                                                                                                                                                                                                                             >  1:244276‑M1/1‑236 (MQ=255)`  
                                                                  `cACTAAAGGAGACACACCATGTTCAAACTGATTAAGAAGTTAGGCCAACTGCTGGTTCGTATGTACAACGTGGAAGCCAAGCGACTGAACGATGAGGCTCGTAAAGAGGCCACACAGTCACGCGCTCTGGCGATTCGCTCCAACGAACTGGCTGACAGTGCATCCACTAAAGTTACCGAGGCTGCCCGTGTGGCAAACCAAGCTCAACAGCTTTCCAAATTCTTTGAGTAAT‑C‑AAtaggagaaatcaatatgatcgtttctgacatcgaagctaacgccctcttagagagcttacctaagt                                                                                                                                                                                                                                            >  1:286941‑M1/1‑235 (MQ=255)`  
                                                                      `aaaGGAGACACACCATGTTCAAACTGATTAAGAAGTTAGGCCAACTGCTGGTTCGTATGTACAACGTGGAAGCCAAGCGACTGAACGATGAGGCTCGTAAAGAGGCCACACAGTCACGCGCTCTGGCGATTCGCTCCAACGAACTGGCTGACAGTGCATCCACTAAAGTTACCGAGGCTGCCCGTGTGGCAAACCAAGCTCAACAGCTTTCCAAATTCTTTGAGTAAT‑CAAAcaggagaaatcaatatgatcgtttctgacatcgaagctaacgccctcttagagagcgtcactaagttac                                                                                                                                                                                                                                         >  1:501537‑M1/1‑232 (MQ=255)`  
                                                                      `aaaGGAGACACACCATGTTCAAACTGATTAAGAAGTTAGGCCAACTGCTGGTTCGTATGTACAACGTGGAAGCCAAGCGACTGAACGATGAGGCTCGTAAAGAGGCCACACAGTCACGCGCTCTGGCGATTCGCTCCAACGAACTGGCTGACAGTGCATCCACTAAAGTTACCGAGGCTGCCCGTGTGGCAAACCAAGCTCAACAGCTTTCCAAATTCTTTGAGTAAT‑C‑AAtaggagaaatcaatatgatcgtttctgacctcgaagctaacgccctcttagagagcgtcactaagttctc                                                                                                                                                                                                                                        >  1:659869‑M1/1‑231 (MQ=255)`  
                                                                         `ggAGACACACCATGTTCAAACTGATTAAGAAGTTAGGCCAACTGCTGGTTCGTATGTACAACGTGGAAGCCAAGCGACTGAACGATGAGGCTCGTAAAGAGGCCACACAGTCACGCGCTCTGGCGATTCGCTCCAACGAACTGGCTGACAGTGCATCCACTAAAGTTACCGAGGCTGCCCGTGTGGCAAACCAAGCCCAACAGCTTTCCAAATTCTTTGAGTAAT‑CAAAcaagagaaatcaatatgatcgtttatgacatcgaagctaaagcactcttagagctgtctcgtatacacatct                                                                                                                                                                                                                                      >  1:630974‑M1/1‑229 (MQ=255)`  
                                                                               `gttataagagacagctgattaagaagttaGGCAAACTGCTGGTTCGTATGTACAACGTGGAAGCCAAGCGACTGAACGATGAGGCTCGTAAAGAGGCCACACAGTCACGCGCTCTGGCGATTCGCTCCAACGAACTGGCTGACAGTGCATCCACTAAAGTTACCGAGGCTGCCCGTGTGGCAAACCAAGCTCAACAGCTTTCCAAATTCTTTGAGTAAT‑C‑AAtaggagaaatcaatatgatcgtttctgacatcgaagctaacgccctcttagagagcgtcactaagttccactgcggggt                                                                                                                                                                                                                               <  1:447806‑M1/287‑80 (MQ=255)`  
                                                                                          `aaaCTGATTAAGAAGTTAGGCCAACTGCTGGTTCGTATGTACAACGTGGAAGCCAAGCGACTGAACGATGAGGCTCGTAAAGAGGCCACACAGTCACGCGCTCTGGCGATTCGCTCCAACGAACTGGCTGACAGTGCATCCACTAAAGTTACCGAGGCTGCCCGTGTGGCAAACCAAGCTCAACAGCTTTCCAAATTCTTTGAGTAAT‑CAAAcaggagaaatcaatatgatcgtttctgacatcgaagctaacgccctcttagagagcgtcactaagttccactgcggggttatctacgac                                                                                                                                                                                                                     <  1:536786‑M1/301‑90 (MQ=255)`  
                                                                                          `aaaCGGATTAAGAAGTTAGGCCAACGGCTGGTTCGTATGTACAACGTGGAAGCCAAGCGACTGAACGATGAGGCTCGTAAAGAGGCCACACAGTCACGCGCTCTGGCGATTCGCTCCAACGAACTGGCTGACAGTGCACCCACTAAAGTTACCGAGGCTGCCCGTGTGGCAAACCAAGCTCAACAGCTTTCCAAATTCTTTGAGTAATCC‑AAcaggagaaatcaatatgatcgtttctgacatcgaagctaacgccctcttagagagcgtcactaatttccactgcggggttatctacgac                                                                                                                                                                                                                     <  1:577820‑M1/301‑90 (MQ=255)`  
                                                                                               `gATTAAGAAGTTAGGCCAACTGCTGGTTCGTATGTACAACGTGGAAGCCAAGCGACTGAACGATGAGGCTCGTAAAGAGGCCACACAGTCACGCGCTCTGGCGATTCGCTCCAACGAACTGGCTGACAGTGCATCCACTAAAGTTACCGAGGCTGCCCGTGTGGCAAACCAAGCTCAACAGCTTTCCAAATTCTTTGAGTAAT‑CAAAtaggagaaatcaatatgatcgtttctgacatcgaagctaacgccctcttagagagcgtcactaagttccactgcggggttatctacgactactc                                                                                                                                                                                                                >  1:441387‑M1/1‑207 (MQ=255)`  
                                                                                                `aTTAAGAAGTTAGGCCAACTGCTGGTTCGTATGTACAACGTGGAAGCCAAGCGACTGAACGATGAGGCTCGTAAAGAGGCCACACAGTCACGCGCTCTGGCGATTCGCTCCAACGAACTGGCTGACAGTGCATCCACTAAAGTTACCGAGGCTGCCCGTGTGGCAAACCAAGCTCAACAGCTTTCCAAATTCTTTGAGTAAT‑C‑AAtaggagaaatcaatatgatcgtttctgacatcgaagctaacgccctcttagagagcgtcactaatttccactgcggggttatctacgacctgtctc                                                                                                                                                                                                              >  1:148687‑M1/1‑205 (MQ=255)`  
                                                                                                    `agaagTTAGGCCACCTGCTGGTTCGTATGTACAACGTGGAAGCCAAGCGACTGAACGATGAGGCTCGTAAAGAGGCCACACAGTCACGCGCTCTGGCGATTCGCTCCAACGAACTGGCTGACAGTGCATCCACTAAAGTTACCGAGGCTGCCCGTGTGGCAAACCAAGCTCAACAGCTTTCCAAATTCTTTGAGTAAT‑C‑AAtaggagaaatcaatatgatcgtttctgacatcgaagctaacgccctcttagagagcgtcactaagttccactgcggggttatctacgactactccaccgc                                                                                                                                                                                                          <  1:591794‑M1/301‑101 (MQ=255)`  
                                                                                                    `agaagTTAGGCCAACTGCTGGTTCGTATGTACAACGTGGAAGCCAAGCGACTGAACGATGAGGCTCGTAAAGAGGCCACACAGTCACGCGCTCTGGCGATTCGCTCCAACGAACTGGCTGACAGTGCATCCACTAAAGTTACCGAGGCTGCCCGTGTGGCAAACCAAGCTCAACAGCTTTCCAAATTCTTTGAGTAAT‑C‑AAtaggagaaatcaatatgatcgtttctgacatcgaagctaacgccctcttagagagcgtcactaagttccactgcggggttatctacgactactccaccgc                                                                                                                                                                                                          <  1:395716‑M1/301‑101 (MQ=255)`  
                                                                                                       `agTTAGGCCAACTGCTGGTTCGTATGTACAACGTGGAAGCCAAGCGACTGAACGATGAGGCTCGTAAAGAGGCCACACAGTCACGCGCTCTGGCGATTCGCTCCAACGAACTGGCTGACAGTGCATCCACTAAAGTTACCGAGGCTGCCCGTGTGGCAAACCAAGCTCAACAGCTTTCCAAATTCTTTGAGTAAT‑C‑AAtaggagaaatcaatatgatcgtttctgacatcgaagctaacgccctcttagagagcgtcactaagttccactgcggggttatctacgactactccaccgctga                                                                                                                                                                                                       >  1:636241‑M1/1‑198 (MQ=255)`  
                                                                                                           `aGGCCTACTGCTGGTGCGTATGTACAACGTGGAAGCCAAGCGACTGAACGATGAGGCTCGTAAAGAGGCCACACAGTCACGCGCTCTGGCGATTCGCTCCAACGAACTGGCTGACAGTGCATCCACTAAAGTTACCGAGGCTGCCCGTGTGGCAAACCAAGCTTAACAGCTTTCCAAATTCTTTGAGTAAT‑C‑AAtaggagaaatcaatatgatcgtttctgacatcgaagctaacgccctcttagagagcgtcactaagttccactgcggggttatctacgactactccaccgctgagtac                                                                                                                                                                                                   <  1:280350‑M1/301‑108 (MQ=255)`  
                                                                                                              `ataagagacagttcgtatgtaCAACGTGGAAGCCAAGCGACTGAACGATGAGGATCGTAAAGAGGCCACACAGGCACGCGCTCTGGCGATTCGCTCCAACGAACTGGCTGACAGTGCATCCACTAAAGTTACCGAGGCTGCCCGTGTGGCAAACCAAGCTCAACAGCTTTCCAAATTCTTTGAGTAAT‑C‑AAtaggagaaatcaatatgatcgtttctgacatcgaagcgaacgccctcttagagagcgtcactaagttccactgcggggttatctacgactactccaccgctgagtacgta                                                                                                                                                                                                <  1:354275‑M1/291‑111 (MQ=255)`  
                                                                                                              `acagctgctggttcGGATGTACAACGTGGAAGCCAAGCGACTGAACGATGAGGCTCGTAAAGAGGCCACACAGTCACGCGCTCTGGCGATTCGCTCCAACTAACTGGCTGACAGTGCATCCACTAAAGTTACCGAGGCTGCACGTGTGGCAAACCAAGCTCAACAGCATTCCAAATTCTTTGAGTAAT‑C‑AAtaggagaaaaaaatatgatcgtttctgacatcgaagctaacgcccgcttagagagcgtcactaagttccactgcggggttatctacgactactccaccgctgagtacgta                                                                                                                                                                                                <  1:602073‑M1/297‑111 (MQ=255)`  
                                                                                                                 `gctcggagatgtgtataagagacaggaagccaagcgactgaacgatgaggCTCGTAAAGAGGCCACACAGTCACGCGCTCTGGCGATTCGCTCCAACGAACTGGCTGACAGTGCATCCACTAAAGTTACCGAGGCTGCCCGTGTGGCAAACCAAGCTCAACAGCTTTCCAAATTCTTTGAGTAAT‑C‑AAtaggagaaatcaatatgatcgtttctgacatcgaagctaacgccctcttagagagcgtcactaagttccactgcggggttatctacgactactccaccgctgagtacgtaagc                                                                                                                                                                                             <  1:585301‑M1/277‑114 (MQ=255)`  
                                                                                                                 `aCTGCTGGTTCGTATGTACAACGTGGAAGCCAAGCGACTGAACGATGAGGCTCGTAAAGAGGCAACACAGTCACGCGCTCTGGCGATTCGCTCCAACGAACTGGCTGACAGTGCATCCACTAAAGTTACCGAGGCTGCCCGTGTGGCAAACCAAGCTCAACAGCTTTCCAAATTCTTTGAGTAAT‑C‑AAtaggagaaatcaatatgatcgtttctgacatcgaagctaacgccctcttagagagcgtcactaagttccactgcggggttatctacgactactccaccgctgagtacgtaagc                                                                                                                                                                                             <  1:190792‑M1/301‑114 (MQ=255)`  
                                                                                                                  `ctgctgGTTCGTATGTACAACGTGGAAGCCAAGCGACTGAACGATGAGGCTCGTAAAGAGGCCACACAGTCACGCGCTCTGGCGATTCGCTCCAACGAACTGGCTGACAGTGCATCCACTAAAGTTAACGAGGCTGCCCGTGTGGCAAACCAAGCTCAACAGCTTTCCAAATTCTTTGAGTAAT‑C‑AAtaggagaaatcaatatgatcgtttctgacatcgaagctaacgccctcttagagagagtcactaagttccactgcggggttatctacgactactcaaccgccgagtacgtaagct                                                                                                                                                                                            >  1:261551‑M1/1‑187 (MQ=255)`  
                                                                                                                  `ctgctgGTTCGTATGTACAACGTGGAAGCCAAGCGACTGAACGATGAGGCTCGTAAAGAGGCCACACAGTCACGCGCTCTGGCGATTCGCTCCAACGAACTGGCTGACAGCGCATCCACTAAAGTTACCGAGGCTGCCCGTGTGGCAAACCAAGCTCAACAGCTTTCCAAATTCTTTGAGTAAT‑C‑AAtaggagaaatcaatatgatcgtttctgacatcgaagctaacgccctcttagagagcgtcactaagttccactgcggggttatctacgactactccaccgctgcgtacgtaagct                                                                                                                                                                                            >  1:122354‑M1/1‑187 (MQ=255)`  
                                                                                                                       `gagacagatgtacaaCGTGGAAGCCAAGCGACTGAACGATGAGGCTCGTAAAGAGGCCACACAGTCACGCGCTCTGGCGATTCGCTCCAACGAACTGGATGACAGTGCATCCACTAAAGTTACCGAGGCTGCCCGTGTGGCAAACCAAGCTCAACAGCTTTCCAAATTCTTTGAGTAAT‑CAAAcaggagaaaccattatgatcgtttctgacatcgaagctaacgccctcttagagagcgtcactaagttccactgcggggttatctacgactactccaccgctgagtacgtaagctaccg                                                                                                                                                                                        <  1:292728‑M1/294‑119 (MQ=255)`  
                                                                                                                        `gTTCGTATGTACAACGTGGAAGCCAAGCGACTGAACGATGAGGCTCGTAAAGAGGCCACACAGTCACGCGCTCTGGCGATTCGCTCCAACGAACTGGCTGACAGTGCATCCACTAAAGTTACCGAGGCTGCCCGTGTGGCAAACCAAGCTCAACAGCTTTCCAAATTCTTTGAGTAAT‑C‑AAtaggagaaagcaatatgatcgtttctgacatcgaagctaacgccctcttagagagcgtcactaagttccactgcggggttatctacgactactccaccgctgagtacgtaagctaccgtc                                                                                                                                                                                      <  1:184733‑M1/301‑121 (MQ=255)`  
                                                                                                                                `gTACAACGTGGAAGCCAAGCGACTGAACGATGAGGCTCGTAAAGAGGCCACACAGTCACGCGCTCTGGCGATTCGCTCCAACGAACTGGCTGACAGTGCATCCACTAAAGTTACCGAGGCTGCCCGTGTGGCAAACCCAGCTCAACAGCTTTCCAAATTCTTTGAGTAAT‑CAAAcaggagaaatcaatatgatcgtttctgacatcgaagctaacgccctcttagagagcgtcactaagttccactgcggggttatctacgactactccaccgctgagtacgtaagctaccgtccgagtga                                                                                                                                                                               >  1:298035‑M1/1‑174 (MQ=255)`  
                                                                                                                                  `aCAACGTGGAAGCCAAGCGACTGAACGATGAGGCTCGTAAAGAGGCCACACAGTCACGCGCTCTGGCGATTCGCTCCAACGAACTGGCTGACAGTGCATCCACTAAAGTTACCGAGGCTGCCCGTGTGGCAAACCAAGCTCAACAGCTTTCCAAATTCTTTGAGTAAT‑C‑AAtaggagaaatcaatatgatcgtttctgacatcgaagctaacgccctcttagagagcgtcactaagttccactgcggggttatctacgactactccaccgctgagtacgtaagctaccgtccgagtgactt                                                                                                                                                                            <  1:129479‑M1/301‑131 (MQ=255)`  
                                                                                                                                  `aCAAAGTGGAAGCCAAGCGACTGAACGATGAGGCTCGTAAAGAGGCCACACAGTCACGCGCTCTGGCGATTCGCTCCAACGAACTGGCTGACAGTGCATCCACTAAAGTTACCGAGGCTGCCCGTGTGGCAAACCAAGCTCAACAGCTTTCCAAATTCTTTGAGTAAT‑C‑AAtaggagaaatcaatatgatcgtttctgacatcgaagctaacgccctcttagagagcgtcactaagttccactgcggggttatctacgactactccaccgctgagtacgtaagctaccgtccgagtgactt                                                                                                                                                                            <  1:67847‑M1/301‑131 (MQ=255)`  
                                                                                                                                       `gTGGAAGCCAAGCGACTGAACGATGAGGCTCGTAAAGAGGCCACACAGTCACGCGCTCTGGCGATTCGCTCCAACGAACTGGCTGACAGTGCATCCACTAAAGTTACCGAGGCTGCCCGTGTGGCAAACCAAGCTCAACAGCTTTCCAAATTCTTTGAGTAAT‑CAAAcaggagaaatcaatatgatcgtttctgacatcgaagctaacgccctcttagagagcgtcactaagttccactgcggggttatctacgactactccaccgctgagtacgtaagctaccgtccgagtgacttcggt                                                                                                                                                                        >  1:159920‑M1/1‑167 (MQ=255)`  
                                                                                                                                             `gCCAAGCGACTGAACGATGAGGCTCGTAAAGAGGCCACACAGTCACGCGCTCTGGCGATTCGCTCCAACGAACTGGCTGACAGTGCATCCACTAAAGTTACCGAGGCTGCCCGTGTGGCAAACCAAGCTCAACAGCTTTCCAAATTCTTTGAGTAAT‑C‑AAtaggagaaatcaatatgatcgtttctgacatcgaagctaacgccctcttagagagcgtcactaagttccactgcggggttatctacgactactccaccgatgagtacgtaagctaccgtccgagtgacttcggtgcgtatc                                                                                                                                                                 <  1:268363‑M1/301‑142 (MQ=255)`  
                                                                                                                                                                `gggcacttaacgtggccacaccgccaccCGCTCTGGCGTTTCGCGCCAACGAACGGGCTGACAGTGCATCCACTAAAGTTACCGAGTCTGCCCGTTTGGC‑AACCAAGCTCAACAGCTTTCCAAATTCTTTGAGTAAT‑C‑AAtaggagaaatcaatatgatcgtttctgacatcgaagctaacgccctcttagagagcgtcactaagttccactgcggggttatctacgactactccaccgctgagtacgtaagctaccgtccgagtgacttcggtgcgtatctggatgcgctggaagccgag                                                                                                                                             <  1:56313‑M1/288‑162 (MQ=255)`  
                                                                                                                                                                              `gCCACACAGTCACGCGCTCTGGCGATTCGCTCCAACGAACTGGCTGACAGTGCATCCACTAAAGTTACCGAGGCTGCCCGTGTGGCAAACCAAGCTCAACAGCTTTCCAAATTCTTTGAGTAAT‑C‑AAtaggagaaatcaatatgatcgtttctgacatcgaagctaacgccctcttagagagcgtcactaagttccactgcggggttatctactactactccaccgctgagtacgtaagctaccgtccgagtgacttcggtgcgtatctggatgcgctggaagccgcggttgcacgaggcg                                                                                                                                >  1:472694‑M1/1‑127 (MQ=255)`  
                                                                                                                                                                                  `aacagTCACGCGCTCTGGCGATTCGCTCCAACGAACTGGCTGACAGTGCATCCACTAAAGTTACCGAGGCTGCCCGTGTGGCAAACCAAGCTCAACAGCTTTCCAAATTCTTTGAGTAAT‑C‑AAtaggagaaatcaatatgatcgtttctgacatcgaagctaacgccctcttagagagcgtcactaagttccactgcggggttatctacgactactccaccgctgagtacgtaagctaccgtccgagtgacttcggtgcgtatctggatgcgctggaagccgaggttgcacgaggcggtct                                                                                                                            <  1:255371‑M1/300‑179 (MQ=255)`  
                                                                                                                                                                                        `gtgggctcggagatgtgtataagagacagctggctgacagtgcatccactaaagttaccGAGGCTGCCCGTGTGGCAAACCAAGCTCAACAGCTTTCCAAATGCTTTGAGTAAT‑CAAAcaggagaaatcaatatgatcgtttctgacatcgaagctaacgccctcttagagagcgtcactaagttccactgcggggttatctacgactactccaccgctgagtacgtaagctaccgtccgagtgacttcggtgcgtatctggatgcgctggaagccgaggttgcacgaggcggtcttattg                                                                                                                       <  1:530841‑M1/272‑184 (MQ=255)`  
                                                                                                                                                                                        `cACGCGCTCTGGCGATTCGCTCCAACGAACTGGCTGACAGTGCATCCACTAAAGTTACCGAGGCTGCCCGTGTGGCAAACCAAGCTCAACAGCTTTCCAAATTCTTTGAGTAAT‑CAAAcaggagaaatcaatatgatcgtttctgacatcgaagctaacgccctcttagagagcgtcactaagttccactgcggggttatctacgactactccaccgctgagtacgtaagctaccgtccgagtgacttcggtgcgtatctggatgcgctggaagccgaggttgcacgaggcggtcttattg                                                                                                                       <  1:178465‑M1/301‑184 (MQ=255)`  
                                                                                                                                                                                        `cACGCGCTCTGGCGATTCGCTCCAACGAACTGGCTGACAGTGCATCCACTAAAGTTACCGAGGCTGCCCGTGTGGCAAACCAAGCTCAACAGCTTTCCAAATTCTTTGAGTAAT‑CAAAcaggagaaatcaatatgatcgtttctgacatcgaagctaacgccctcttagagagcgtcactaagttccactgcggggttatctacgactactccaccgctgagtacgtaagctaccgtccgagtgacttcggtgcgtatctggatgcgctggaagccgaggttgcacgaggcggtcttattg                                                                                                                       <  1:232312‑M1/301‑184 (MQ=255)`  
                                                                                                                                                                                        `atgtgtataagagacagcgctccaacgaactggctGACAGTGCATCCACTAAAGTTACCGAGGCTGCCCGTGTGGCAAACCAAGCTCAACAGCTTTCCAAATTCTTTGAGTAAT‑CAAAcaggagaaatcaatatgatcgtttctgacatcgaagctaacgacctcttagagagcgtcactaagttccactgcggggttatctacgactactccaccgctgagtacgtaagctaccgtccgagtgacttcggtgcgtatctggatgcgctggaagccgaggttgcacgaggcggtcttattg                                                                                                                       <  1:115782‑M1/284‑184 (MQ=255)`  
                                                                                                                                                                                        `aacacactctggcgatTCGCTCCAAGGAACTGGCTGACAGTGCATCCACTAAAGGTACCGAGGCTGCCCGTGTGGCAAACCAAGCTCAACAGCTTTCCAAATTCTTTGAGTAAT‑CAAAcaggagaaatcaatatgatcgtttctgacatcgaagctaacgccctcttagagagcgtcactaagttccactgcggggttatctacgactactccaccgctgagtacgtaagctaccgtccgagtgacttcggtgcgtatctggatgcgctggaagccgaggttgcacgaggcggtcttattg                                                                                                                       <  1:642391‑M1/295‑184 (MQ=255)`  
                                                                                                                                                                                            `cgcTCTGGCGATTCGCTCCAACGAACTGGCTGACAGTGCATCCACTAAAGTTACCGAGGCTGCCCGTGTGGCAAACCAAGCTCAACAGCTTTCCAAATTCTTTGAGTAAT‑C‑AAtaggagaaatcaatatgatcgttactgacatcgaagctaacgccctcttagagagcgtcactaagttccactgcggggttatctacgactactccaccgctgagtacgtaagctaccgtccgagtgacttcggtgcgtatctggatgcgctggaagccgaggttgcacgaggcggtcttattgtgttc                                                                                                                  >  1:327981‑M1/1‑113 (MQ=255)`  
                                                                                                                                                                                              `ctctGGCGATTCGCTCCAACGAACTGGCTGACAGTGCATCCACTAAAGTTACCGAGGCTGCCCGTGTGGCAAACCAAGCTCAACAGCTTTCCAAATTCTTTGAGTAAT‑C‑AAtaggagaaatcaatatgatcgtttctgacatcgaagctaacgccctcttagagagcgtcactaagttccactgcggggttatctacgactactccaccgctgagtacgtaagctaccgtccgagtgacttcggtgcgtatctggatgcgctggaagccgaggttgcacgaggcggtcttattgtgttccc                                                                                                                >  1:15455‑M1/1‑111 (MQ=255)`  
                                                                                                                                                                                               `tctGGCGATTCGCTCCAACAAACTGGCGGACAGTGCATCCACTAAAGTTACCGAGGCTGCCCGTGGGGCAAACCAAGCTTAACAGCTTTCCAAAGTCTTTGAGTAAT‑C‑AAtaggagaaatcaatatgatcgtttctgacatcgaagctaacgccctcttagagagcgtcactaagtaccactgcggggttatctacgactactccaccgctgagtacgtaagctaccgtccgagtgacttcggtgcgtatctggatgcgctggaagccgaggttgcactaggcggtcttattgtgttccac                                                                                                               <  1:237422‑M1/301‑192 (MQ=255)`  
                                                                                                                                                                                                 `tGGCGCTTCGCTCCAACGAACTGGCTGTCAGTGCATCCACTAAAGTTACCGAGGCTGCCCGTGTGGCAAACCAAGCTCAACAGCTTTCCAAATTCTTTGAGTAAT‑C‑AAtaggagaaatcaatatgatcgtttctgacatcgaagctaacgccctcttagagagcgtcactaagttccactgcggggttatctacgactactccaccgctgagtacgtaagctaccgtccgagtgacttcggtgcgtatctggatgcgctggaagccgaggttgcacgaggcggtcttattgtgttccacaa                                                                                                             <  1:220727‑M1/301‑194 (MQ=255)`  
                                                                                                                                                                                                  `ggCGATTCGCTCCAACGAACTGGCTGACAGTGCATCCACTAAAGTTACCGAGGCTGCACGTGTGGCAAACCAAGCTCAACAGCTTTCCAAATTCTTTGAGTAAT‑C‑AAtaggagaaatcaatatgatcgtttctgacatcgaagctaacgccctcttagagagcgtcactaagttcaactgcggggttatctacgactactccaccgctgagtacgtaagctaccgtccgagtgacttcggtgcgtagctggatgcgctggaagccgagtttgcacgaggcggtcttattttgttccacaac                                                                                                            >  1:369850‑M1/1‑107 (MQ=255)`  
                                                                                                                                                                                                           `cTCCAACGAACTGGCTGACAGTGCATCCACTAAAGTTACCGAGGCTGCCCGTGTGGCAAACCAAGCTCAACAGCTTTCCAAATTCTTTGAGTAAT‑C‑AAtaggagaaatcaatatgatcgtttctgacatcgaagctaactccctcttagagagcgtcactaagttccactgcgggggttatctacgactactccaccgctgagtacgtaagctaccgtccgagtgacttcggtgcgtatctggatgcgctggaagccgaggttgcacgaggcggtcttattgtgctgtctcttatacacat                                                                                                   >  1:509073‑M1/1‑98 (MQ=255)`  
                                                                                                                                                                                                             `ccAACGAACTGGCTGACAGTGCATCCACTAAAGTTACCGAGGCTGCCCGTGTGGCAAACCAAGCTCAACAGCTTTCCAAATTCTTTGAGTAAT‑CAAAcaggagaaatcaatatgatcgtttctgacatcgaagctaacgctctcttagagagcgtcactaagttccactgcggggttatctacgactactccaccgctgagtacgtaagctaccgtccgagtgacttcggtgcgtatctggatgcgctggaagccgaggttgcacgaggcggtcttattgtgttccctgtctcttatacac                                                                                                  >  1:407540‑M1/1‑97 (MQ=255)`  
                                                                                                                                                                                                                 `agaACTGGCTGACAGTGCATCCACTAAAGTTACCGAGGCTGCCCGTGTGGCAAACCAAGCTCAACAGCTTTCCAAATTCTTTGAGTAAT‑CAAAcaggagaaatcaatatgatcgtttctgacatcgaagctaacgccctcttagagagcgtcactaagttccactgcggggttatctacgactactccaccgctgagtacgtaagctaccgtccgagtgacttcggtgcgtatctggatgcgctggaagccgaggttgcacgaggcggtcttattgtgttccacaacggtcacaagtatga                                                                                              <  1:419633‑M1/300‑209 (MQ=255)`  
                                                                                                                                                                                                                        `gCTGACAGTGCATCCACTAAAGTTACCGAGGCTGCCCGTGTGGCAAACCAAGCTCAACAGCTTTCCAAATTCTTTGAGTAAT‑C‑AAtaggagaaatcaatatgatcgtttctgacatcgaagctaacgccctcttagagagcgtcactaagttccactgcggggttatctacgactactccaccgctgagtacgtaagctaccgtccgagtgacttcggtgcgtatctggatgcgctggaagccgaggttgcacgaggcggtcttattgtgttccacaacggtcacaagtatgacgttcctg                                                                                      <  1:490878‑M1/301‑217 (MQ=255)`  
                                                                                                                                                                                                                           `gtataagagacagctaaagttaccgagGCTGCCCGTGTGGCAAACCAAGCTCAACAGCTTTGCAAATTCTTTGAGTAAT‑C‑AAtaggagaaatcaatatgatcgtttctgacatcgaagctaacgcccgcttagagagcgtcactaagttccactgcggggttatctacgactactccaccgctgagtacgtaagctaccgtccgagtgacttcggtgcgtatctggatgcgctggaagccgaggttgcacgaggcggtcttattgtgttccacaacggtaacaagtatgacgttcctgcat                                                                                   <  1:360493‑M1/288‑220 (MQ=255)`  
                                                                                                                                                                                                                           `gACAGTGCATCCACTAAAGTTACCGAGGCTGCCCGGGTGGCAAACCAAGCTCAACAGCTTTCCAAATTCTTTGAGTAAT‑C‑AAtaggagaaatcaatatgatcgtttctgacatcgaagctaacgccctcttagagagcgtcactaagttccactgcggggttatctacgactactccaccgctgagtacgtaagctaccgtccgagtgacttcggtgcgtatctggatgcgctggaagccgaggttgcacgaggcggtcttattgtgttccacaacggtcacaagtatgacgttcctgcat                                                                                   <  1:796‑M1/301‑220 (MQ=255)`  
                                                                                                                                                                                                                                      `cACTAAAGTTACCGAGGCTGCCCGTGTGGCAAACCAAGCTCAACAGCTTTCCAAATTCTTTGAGTAAT‑CAAAcaggagaaatcaatatgatcgtttctgacatcgaagctaacgccctcttagagagcgtcactaagttccactgcggggttatctacgactactccaccgctgagtacgtaagctaccgtccgagtgacttcggtgcgtatctggatgcgctggaagccgaggttgcacgaggcggtcttattgtgttccacaacggtcacaagtatgacgttcctgcatttaccctgtc                                                                         >  1:203834‑M1/1‑72 (MQ=255)`  
                                                                                                                                                                                                                                            `aGTTACCGAGGCTGCCCGTGTGGCAAACCAAGCTCAACAGCTTTCCAAATTCTTTGAGTAAT‑CAAAcaggagaaatcaatatgatcgtttctgacatcgaagctaacgccctcttagagagcgtcactaagttccactgcggggttatctacgactactccaccgctgagtacgtaagctaccgtccgagtgacttcggtgcgtatctggatgcgctggaagccgaggttgcacgaggcggtcttattgtgttccacaacgttcacaagtatgacgttcctgcattgaccaaactggcaaa                                                                   >  1:322582‑M1/1‑66 (MQ=255)`  
                                                                                                                                                                                                                                             `gtgaccgGGGCTGCCCGGGTGGAAAACCAAGCTCAACAGCTTTCCAAATTCTTTGAGTAAT‑C‑AAtaggagaaagcaatatgatcgtttctgacatcgaagctaacgccctcttagagagcgtcactaaggcccactgcgggggtatctacgactactccaccgctgagtacgtaagctaccgtccgagtgacttcggtgcgtatctggatgcgctggaagccgaggttgcacgaggcggtcttattgtgttccacaacggtcacaagtatgacgttcctgcattgaccaaactggcaaagt                                                                 <  1:364427‑M1/298‑238 (MQ=255)`  
                                                                                                                                                                                                                                              `ttACCGAGGCTGCCCGTGTGGCAAACCAAGCTCAACAGCTTTCCAAATTCTTTGAGTAAT‑C‑AAtaggagaaatcaatatgatcgtttctgacatcgaagctaacgccctcttagagagcgccactaagttccactgcggggttatctacgactactccaccgctgagtacgtaagctaccgtccgagtgacttcggtgcgtatctgtatgcgctggaagcagaggttgcacgaggcggtcttcttgtgttccacaacggttacaagtatgtcgttccccacttgccccacctgtcaaagat                                                                >  1:648576‑M1/1‑63 (MQ=255)`  
                                                                                                                                                                                                                                               `tACCGAGGCTGCCCGTGTGGCAAACCAAGCTCAACAGCTTTCCAAATTCTTTGAGTAAT‑CAAAcaggagaaatcaatatgatcgtttctgacatcgaagctaacgccctcttagagagcgtcactaagttccactgcggggttatctacgactactccaccgctgagtacgtaagctaccgtccgagtgacttcggtgcgtatctggatgcgctggaagccgaggttgcacgaggcggtcttattgtgttccacaacggtcacaagtatgacgttcctgcattgaccaaactggcaaagtt                                                                >  1:515885‑M1/1‑63 (MQ=255)`  
                                                                                                                                                                                                                                                             `gtgtGGCAAACCACGCTCAACAGCTTTCCAAATTCTTTGAGTAAT‑C‑AAtaggagaaatcaatatgatcgtttctgacatcgaagctaacgccctcttagagagcgtcactaagttccactgcggggttatctacgactactccaccgctgagtacgtaagctaccgtccgagtgacttcggtgcgtatctggatgcgctggaagccgaggttgcacgaggcggtcttattgtgttccacaatggtcacaattatgacgttcctgcattgaccaaactggcaaagttgcaattgaaccgaga                                                 >  1:435678‑M1/1‑48 (MQ=255)`  
                                                                                                                                                                                                                                                                `tgGCAAACCAAGCTCAAAAGATTTCCAAATTCGTGGAGTAAT‑CAAAcaggagaaatcaatatgatcggttctgacatcgaagcgaacaccctcgtagagagcgtcactaagttccacggcggggttatctacgactactccaccgctgagtacgtaagctaccgtccgagtgacttcggtgcgtatctggatgcgctggaagccgaggttgcacgaggcggtcttattgtgttccacaacggtcacaagtatgacgttcctgcattgaccaaactggcaaagttgcaattgaaccgagagt                                               <  1:134564‑M1/301‑256 (MQ=255)`  
                                                                                                                                                                                                                                                                                    `cTTTCAAAATTATTTGAGTAAT‑CAAAcaggagaaatcaatatgatcgtttctgacatcgaagctaacgccctcttagagagcgtcactaagttccactgcggggttatctacgactactccaccgctgagtacgtaagctaccgtccgagtgacttcggtgcgtatctggatgcgctggaagccgaggttgcacgaggcggtcttattgtgttccacaacggtcacaagtatgacgttcctgcattgaccaaactggcaaagttgcaattgaaccgagagttccaccttcctcgtgagaac                           <  1:453803‑M1/301‑276 (MQ=255)`  
                                                                                                                                                                                                                                                                                       `tCCAAATTCTTTGAGTAAT‑CAAAcaggagaaatcaatatgatcgtttctgacatcgaagctaacgccctcttagagagcgtcactaagttccactgcggggttatctacgactactccaccgctgagtacgtaagctaccgtccgagtgacttcggtgcgtatctggatgcgctggaagccgaggttgcacgaggcggtcttattgtgttccacaacggtcacaagtatgacgttcctgcattgaccaaactggcaaagttgcaattgaacctagagttccaccttcctcgtgagaactgt                        >  1:458264‑M1/1‑23 (MQ=255)`  
                                                                                                                                                                                                                                                                                         `cAAATTCTGTGAGTAAT‑CAAAcaggagaaattaatatgctcgtttataacatcgaagctaaagccctcacagagagcgtcactaagttccactgcggggttatctacgactgctccaccgctgagtacgtaagctaccgtccgagtgacttcgatgcgtatctggatgcgctggaagccgaggttgcacgaggcggtcttaatgtggtccacaacggtcacaagtatgacgtgcatgcattgaccaaactggcaaagttgcaattgaaccgagagttccaccttcctcgtgagagctgtat                      <  1:615278‑M1/301‑281 (MQ=255)`  
                                                                                                                                                                                                                                                                                             `ttCTTTGAGTAAT‑CAAAcaggagaaatcaatatgatcgtttctgacatcgaagctaacgccctcttagagagcgtcactaagttccactgcggggttatctacgactactccaccgctgagtacgtaagctaccgtccgagtgacttcggtgcgtatctggatgcgctggaagccgaggttgcacgaggcggtcttattgtgttccacaacggtcacaagtatgacgttcctgcattgaccaacctggcaaagttgcaattgaaccgagagttccaccttcctcgtgagaactgtattgac                  >  1:551739‑M1/1‑17 (MQ=255)`  
                                                                                                                                                                                                                                                                                             `ttCTTTGAGTAAT‑CAAAcaggagaaatcaatatgatcgtttctgacatcgaagctaacgccctcttagagagcgtcactaagttccactgcggggttatctacgactactccaccgctgagtacgtaagctaccgtccgagtgacttcggtgcgtatctggatgcgctggaagccgaggttgcacgaggcggtcttattgtgttccacaacggtcacaagtatgacgttcctgcattgaccaaactggcaaagttgcaattgaaccgagagttccaccttcctcgtgagaactgtattgac                  >  1:209308‑M1/1‑17 (MQ=255)`  
                                                                                                                                                                                                                                                                                             `agagacaggtaat‑c‑aataggagaaatcaatatgatcgtttctgacatcgaagctaacgccctcttagagagcgtcactaagttccactgcggggttatctacgactactccaccgctgagtacgtaagctaccgtccgagtgacttcggtgcgtatctggatgcgctggaagccgaggttgcacgaggcggtcttattgtgttccacaacggtcacaagtatgacgttcctgcattgaccaaactggcaaagttgcaattgaaccgagagttccaccttcctcgtgagaactgtattgaca                 <  1:620357‑M1/293‑286 (MQ=255)`  
                                                                                                                                                                                                                                                                                              `tCTATGAGTAAT‑C‑AAtaggagtaatcaagaggatcgtttctgacagcgaagctaacgccctcttagagagcgtcactaagttccactgcggggttatctacgactactccaccgctgagtacgtaagctaccgtccgagtgacttcggtgcgtatctggatgcgctggaagccgatgttgcacgaggcggtcttattgtgttccacaacggtcacaagtatgacgttcctgcattgaccaaactggcaaagttgcaattgaaccgagagttccaccttcctcgtgagaactgtattgacac                <  1:666847‑M1/301‑287 (MQ=255)`  
                                                                                                                                                                                                                                                                                              `aagagacagaat‑c‑aataggagaaatcaatatgatcgtttctgacatcgaagctaacgccctcttagagagcgtcactaagttccactgcggggttatctacgacgactccaccgctgagtacgtaagctaccgtccgagtgacttcggtgcgtatctggatgcgctggaagccgaggttgcacgaggcggtcttattgtgttccacaacggtcacaagtatgacgttcctgcattgaccagactggcaaagttgcaattgaaccgagagttccacctacctcgtgagaactgtattgacac                <  1:249232‑M1/292‑287 (MQ=255)`  
                                                                                                                                                                                                                                                                                                `tttGAGTAAT‑CAAAcaggagaaaccattatgatcgtttctgacatcgaagctaacgccctcttagagagcgtcactaagttccacggcggggttatctacgactactccaccgctgagtacgtaagctaccgtccgagtgacttcggtgcgtatctggatgcgctggaagccgaggttgcacgaggcggtcttattgtgttccacaacggtcacaagtatgacgttcctgcattgaccaaactggcaaagttgcaattgaacctagagttccaccttcctcgtgagaactgtattgacacc               <  1:105401‑M1/301‑288 (MQ=255)`  
                                                                                                                                                                                                                                                                                                   `gAGTAAT‑C‑AAtaggagaaatcaatatgatcgtttctgacatcgaagctaacgccctcttagagagcgtcactaagttccactgcggggttatctacgactactccaccgctgagtacgtaagctaccgtccgagtgacttcggtgcgtatctggatgcgctggaagccgaggttgcacgaggcggtcttattgtgttccacaacggtcacacgtatgacgttcctgcattgaccaaactggcaaagttgcaattgaaccgagagttccaccttcctcgtgagaaatgtattgacacctgtc           >  1:142292‑M1/1‑10 (MQ=255)`  
                                                                                                                                                                                                                                                                                                     `gTAAT‑C‑AAtaggagaaatcaatatgatcgtttctgacatcgaagctaacgccctcttagagagcgtcactaagttccactgcggggttatctacgactactccaccgctgagtacgtaagctaccgtccgagtgacttcggtgcgtatctggatgcgctggaagccgaggttgcacgaggcggtattattgtgttccacaacggtcacaagtatgacgttcctgcattgaccaaactggcaaagttgcaattgaaccgagagttccaccttcctcgtgagaactgtattgacacccttgtg         >  1:340755‑M1/1‑8 (MQ=255)`  
                                                                                                                                                                                                                                                                                                      `tAAT‑C‑AAtaggagaaataaatatgatcgtttcagacatcgaagctaacgccctcttagagagcgtcactaagttccactgaggtgttatctacgactactccaccgctgagtacgtaagctaccgtccgagtgacttcggtgcgtatctggatgcgctggaagccgagtttgcacgaggcggtcttattgtgttccacaacggtcacaagtatgacgttcctgaattgaccaaactggcaaagttgcaattgaaccgagagttccaccttcctcgtgagaactgtattgacacccttgtgt        <  1:305067‑M1/301‑295 (MQ=255)`  
                                                                                                                                                                                                                                                                                                       `cagataaataggagaaatcaatatgatcgtttctgacatcgaagctaacgccctctgagagagcgtcactaagttccactgcggggttatctacgactactccaccgctgagtacgtaagctaccgtcctagtgacttcggtgcgtatctggatgcgctggaagccgaggttgcacgaggcggtcttattgtgttccacaacggtcacaagtatgacgttcctgcattgaccaaactggcaaagttgcaattgaaccgagagttccaccttcctcgtgagaactgtattgacacccttgtg         <  1:62525‑M1/295‑294 (MQ=255)`  
                                                                                                                                                                                                                                                                                                       `acagc‑aataggagaaatcaatatgatcgtttctgacatcgaagctaacgccctcttagagagcgtcactcagttccactgcggggttatctacgactactccaccgctgagtacgtaagctaccgtccgagtgacttcggtgcgtatctggatgcgctggaagccgaggttgcacgaggcggtcttattgtgttccacaacggtcacaagtatgacgttccttcattgaccaaactggcaaagttgcaattgaaccgagagttccaccttcctcgtgagaactgtattgacacccttgtgt        <  1:495263‑M1/297‑295 (MQ=255)`  
                                                                                                                                                                                                                                                                                                         `t‑C‑AAtaggagaaatcaatatgatcgtttctgacatcgaagctaacgccctcttagagagcgtcactaagttccacttcggggttatctacgactactccaccgctgagtacgtaagctaccgtccgagtgacttcggtgcgtatctggatgcgctggaagccgaggttgcacgaggcggtcttattgtgttccacaacggttacaagtatgacgttcctgcattgaccaaactggcaaagttgcaatttaaccgagagttccaccttcctcgtgagaacctgtctcttatacacatctccc     >  1:74492‑M1/1‑4 (MQ=255)`  
                                                                                                                                                                                                                                                                                                           `c‑AAtaggagaaatcaatatgatcgtttctgacatcgaagctaacgccctcttagagagcgtcactaagttccactgcggggttatctacgactactccaccgctgagtacgtaagctaccgtccgagtgacttcggtgcgtatctggatgcgctggaagccgaggttgcacgaggcggtcttattgtgttccacaacggtcacaagtatgacgttcctgcattgaccaaactggcaaagttgcaattgaaccgagagttccaccttcctcgtgagaactgtattgatactgcttcttataa    >  1:226671‑M1/1‑3 (MQ=255)`  
                                                                                                                                                                                                                                                                                                             `aataggagaaatcaagatgatcctttctgacatcgaatctaacgccctcttagagaccggcactcacttcccctgcggagttatctacgcctactccaccgctgagtacgtaagctaccgtccgagtgacttcggtgcgtatctggatgcgctggaagccgatgttgcacgaggcggtcttattgtgttccacaacggtcacaagtatgacgttcctgcattgaccaaactggcaaagttgcaattgaaccgagagttccaccttcctcgtgagaactgtattgacacccttgtgttgtca   <  1:596382‑M1/301‑300 (MQ=255)`  
                                                                                                                                                                                                                                                                                                              `ataggagaaatcaatatgatcgtttctgacatcgaagctaacgccctcttagagagcgtcactaagttccactgcggggttatctacgactactccaccgctgagtacgtaagctaccgtccgagtgacttcggtgcgtatctggatgcgctggaagccgaggttgcacgaggcggtcttattgtgttccacaacggtcacaagtatgacgttcctgcattgaccaaactggcaacgttgcaattgaaccgagagttccaccttcctcgtgagaactgtattgccacccttgtgttgtcac  >  1:520696‑M1/1‑1 (MQ=255)`  

                                                                                                                                                                                                                                                                                                              `|`                                                                                                                                                                                                                                                                                                              
`GGATTCTTGATGACTTTCCAGACGACTACGAGAAGTTTCGCTGGAGAGTCCCATTCTAATACGACTCACTAAAGGAGACACACCATGTTCAAACTGATTAAGAAGTTAGGCCAACTGCTGGTTCGTATGTACAACGTGGAAGCCAAGCGACTGAACGATGAGGCTCGTAAAGAGGCCACACAGTCACGCGCTCTGGCGATTCGCTCCAACGAACTGGCTGACAGTGCATCCACTAAAGTTACCGAGGCTGCCCGTGTGGCAAACCAAGCTCAACAGCTTTCCAAATTCTTTGAGTAAT‑C‑AAACAGGAGAAACCATTATGTCTAACGTAGCTGAAACTATCCGTCTATCCGATACAGCTGACCAGTGGAACCGTCGAGTCCACATCAACGTTCGCAACGGTAAGGCGACTATGGTTTACCGCTGGAAGGACTCTAAGTCCTCTAAGAATCACACTCAGCGTATGACGTTGACAGATGAGCAAGCACTGCGTCTGGTCAATGCGCTTACCAAAGCTGCCGTGACAGCAATTCATGAAGCTGGTCGCGTCAATGAAGCTATGGCTATCCTCGACAAGATTGATAACTAAGAGTGGTATCCTCAA  >  NC_001604.1/13268‑13868`

  

Alignment Legend

Aligned base mismatch/match (shaded by quality score): `ATCG/ATCG` `< 3 ≤` `ATCG/ATCG` `< 10 ≤` `ATCG/ATCG` `< 22 ≤` `ATCG/ATCG` `< 34 ≤` `ATCG/ATCG` `< 38 ≤` `ATCG/ATCG`

Unaligned base: `atcg`    Masked matching base: `atcg`    Alignment gap: `‑`    Deleted base: `‑`
